# Polyamine-Related Gene Families Identification and Regulatory Effects on Early Somatic Embryogenesis via Modulating Gene Expressions and Hormone Levels in *Ginkgo biloba*

**DOI:** 10.3390/plants15111617

**Published:** 2026-05-25

**Authors:** Jingjing Di, Wenyan Ge, Ying Chen, Yuchen Hu, Yichen Lu, Hao Cai

**Affiliations:** Co-Innovation Center for Sustainable Forestry in Southern China, College of Life Sciences, Nanjing Forestry University, Nanjing 210037, China

**Keywords:** *Ginkgo biloba* L., polyamines, genome-wide analysis, gene expression, embryogenic callus, early somatic embryogenesis

## Abstract

Polyamines (PAs) play critical roles in plant growth, somatic embryogenesis (SE), etc. Previous studies have demonstrated that exogenous PAs could promote SE in plants. However, the effects of PAs on *Ginkgo biloba* L. SE are still unknown, especially in the switch from the initial callus (IC) to the embryogenic callus (EC) stage or to the globular embryo (GE) stage. This work identified 34 genes involved in PAs metabolism in *G. biloba* using genome-wide analyses. These genes were clustered into six families and found to be unevenly distributed across 11 of the 12 chromosomes on the plant. These families contain 539 cis-acting elements that mainly respond to phytohormones, abiotic stress, meristem expression, etc. RNAseq analysis revealed that the expression of *GbADC2*, *GbSAMDC2*, *GbSPMS1*, *GbCuAO1* and *3*, and *GbPAO3*, *8*, *6* and *13* genes in *G. biloba* were higher in the GE stage than in the IC stage. In addition, 1.0 mg·L^−1^ spermine (Spm3) could promote the conversion of IC to EC, while 0.01 mg·L^−1^ putrescine (Put1) could facilitate the transition from IC to EC and then to GE. During the conversion of IC to EC or to GE, higher levels of abscisic acid (ABA), superoxide dismutase (SOD), and peroxidase (POD) and lower levels of indole-3-acetic acid (IAA), gibberellin (GA3), and zeatin (ZT) were observed; concurrently, the H_2_O_2_ level was also observed to be high. Gene expressions of *GbSPMS2*, *GbCuAO3*, and *GbPAO6* and *8* were upregulated, while *GbADC2* expression was downregulated in the EC or GE stages under Spm3- or Put1- treatment. These results illustrate that exogenous PAs might alter the levels of the endogenous polyamine pool and lead to H_2_O_2_ production, which caused a certain oxidative stress. However, SOD and POD balanced H_2_O_2_ production and maintained homeostasis. The PAs–H_2_O_2_–ABA module might coordinate the regulation of early in of *G. biloba*. These relations were discussed in this work. These findings provide a foundation for comprehending the roles of PAs gene families in the key nodes of early SE in *G. biloba*.

## 1. Introduction

Polyamines (PAs) are low-molecular-weight, aliphatic, nitrogen-containing bases, which comprise three to fifteen straight-chain carbon atoms and two or more amino groups. They are widely present in both prokaryotes and eukaryotes, exhibit strong biological activity, and are considered as a new kind of phytohormone [1,2,3,4]. Putrescine (Put), spermidine (Spd) and spermine (Spm) are three important PAs that have been the focus of recent reports. Polyamines are involved in numerous molecular processes through their interaction with signaling metabolites (H_2_O_2_, NO), aminobutyric acid (GABA), and phytohormones such as auxin, abscisic acid, gibberellins, ethylene, etc., as well as in nitrogen metabolism [5,6]. Consequently, it has been established that PAs play important roles in biotic and abiotic stresses, somatic embryogenesis, breaking dormancy, postharvest processes, and secondary metabolism [7,8,9].

In the PAs biosynthetic pathway, putrescine is the central product: it serves as the precursor for the synthesis of spermidine (Spd) and spermine (Spm). First, arginine or ornithine undergoes decarboxylation by either arginine decarboxylase (ADC) or ornithine decarboxylase (ODC), generating Put. Another important substrate for PA synthesis is S-adenosylmethionine (SAM), which can be converted into decarboxylated S-adenosylmethionine (dcSAM) by S-adenosylmethionine decarboxylase (SAMDC). Secondly, Put and dcSAM are converted into Spd by spermidine synthase (SPDS). Finally, Spd and dcSAM are converted into Spm by spermine synthase (SPMS) [3,4,5,10,11,12]. In the catabolism of polyamines, copper-containing ammonia oxidase (CuAO_S_) are responsible for the oxidation of Put, while polyamine oxidases (PAOs) are responsible for the oxidation of Spd or Spm, releasing H_2_O_2_ as a signaling molecule [3,13,14,15].

Somatic embryogenesis (SE) is a process in which somatic cells undergo dedifferentiation to acquire stem cell characteristics and subsequently develop into somatic embryos [16,17]. Somatic embryo formation is an effective tool for the large-scale propagation of plants with excellent genes and rare species in a rapid and efficient manner. It is also an ideal model system for studying somatic reprogramming and cell totipotency, and for clarifying the molecular mechanisms involved [18]. Studying somatic embryogenesis pathways and mechanisms could provide new insights into cell totipotency, regeneration and differentiation [19]. The formation process of plant somatic embryos primarily involves six stages: initial callus (IC), embryogenic callus (EC), globular embryo (GE), heart-shaped embryo (HE), torpedo embryo (TE) and cotyledon embryo (CE). During SE induction, two distinct types of calluses are generated: non-embryogenic callus (NEC) and EC. The NEC is comprised of disorganizing cells, small nuclei and low cytoplasm. These cultures cannot be converted to SE [20]. However, the EC exhibits visible nuclei, dense cytoplasmatic cells, and differentiated cell masses. It has the ability to develop into a somatic embryo [21,22].

Many studies have shown that PAs are also involved in regulating SE development. Maintaining PAs homeostasis is considered to be the premise of SE formation. Put and Spd levels were significantly elevated in carrot embryogenic medium [23]. Put levels increased 27–32-fold in petiole-derived tissue cultures and decreased sharply upon transferring the cultures to an embryo differentiation medium [24]. The distribution and the ratio of PAs were correlated with defined stages of embryogenesis and have been identified as developmental markers of embryogenesis [25]. For example, the level of Put was dominant in somatic embryos prior to germination, while Spd/Put and Spm/Put ratios increased significantly during somatic embryo development compared to the pro-embryogenic stage in *Picea rubens* [26]. Stress treatments such as high temperature led to an elevation of the endogenous Put levels in radiata pine somatic embryos [27].

With the addition of exogenous polyamines into embryo differentiation medium, many research works have evidenced that PAs were indeed involved in somatic embryogenesis [28,29,30]. For instance, adding 10 μM Spd was found to improve the formation of somatic embryo in Scots pine (*Pinus sylvestris* L.) [29]. The embryogenic structures (CE stage) showed an enhancement by four- to five-fold when supplemented with 0.1–1.0 mM Spd in embryogenic callus medium [31]. Adding 1 mM Put and 5 mM a-difluoromethyl ornithine (DFMO) to the embryo-inducing medium enhanced somatic embryogenesis. Meanwhile, the levels of endogenous polyamines (especially Spm) increased remarkably in the globular embryos of *Citrus sinensis* [30]. Adding Put to the regeneration medium increased the number of plants and embryos obtained from carrot cultivars [32]. Put (1.0 mM) induced the highest number of somatic embryos and shoots in indica rice cultivars [33]. Supplementing the medium with 20 mg.L^−1^ Put obtained 106 somatic embryos per 250 mg of callus, and a more than two- to three-fold increase in somatic embryos of sugarcane (*Saccharum*) [34]. The number of secondary embryos increased by 375% and embryo quality improved when 0.4 mg.L^−1^ Spd was supplemented to the basal medium of *Quercus suber* L. [35].

One mechanism by which exogenous polyamines regulate somatic embryogenesis is through altering the endogenous PAs content. The production of somatic embryos increased significantly, while the endogenous Spd level had transiently accumulated when Spd was applied to the initiation medium of *Panax ginseng* [28]. Treatment with 10–500 μM Put significantly increased the endogenous Put levels and reduced endogenous Spd levels in the treated embryogenic suspensor mass of *Picea abies* [36]. The same result was also observed in the SE of *Ocotea catharinensis.* Put treatment increased the number of GEs, while Spd and Spm treatments promoted CE formation. Meanwhile, high levels of endogenous PAs were observed at the GE stage [37].

Another mechanism by which exogenous polyamines (PAs) promote somatic embryogenesis is through regulating changes in endogenous hormones and antioxidant systems. For instance, 1.0 mM of both Put and Spm were found to enhance the growth of embryogenic cultures and the endogenous levels of indole-3-acetic acid (IAA) and abscisic acid (ABA) in *Araucaria angustifolia* [38]. Treatment with Spd and Spm stimulated the release of nitric oxide (NO) from somatic embryos of *Ocotea catharinensis* [37]. In carrot embryogenic cell lines, the activities of ADC and ODC first rose significantly and peaked during the GE stage, then decreased during the TE and CE stages [39]. Adding 0.01 mM Spd to the MS medium promoted the formation of somatic embryo from leaf and petiole explants, and improved somatic embryo quality at the CE stage. Furthermore, Spd enhanced the activity of antioxidant enzymes such as catalase (CAT), superoxide dismutase (SOD), etc., and agitated the fluctuations of endogenous hormones [40].

Differences in the expression of the polyamine synthase gene affect the process of embryonic development. During the transformation period of ECs into GEs (globular embryos) in *Dimocarpus longan*, the expression levels of *DlADC2*, *DlODC*, *DlSPDS1A*, *DlCuAOB* and *DlPAO4B* were upregulated, while those of *DlSAMDC2* and *DlSPMS* were downregulated, following the addition of Put and Spm [12]. Studies have shown that GABA (a PAs metabolite) stimulates the higher expression of *LhWUS*, *LhCLV3* and *LhWOX2*, as well as an earlier *LhCLV3* expression peak. These changes in gene expression might underlie the accelerated somatic embryo induction in *Liriodendron hybrid* [41]. In the somatic embryo aggregates of *Vitis vinifera* L., free Put levels peaked significantly in the second week of culture, while free Spd levels remained low despite the expression of *VvADC*, *VvSPDS2* and *VvPAO* being upregulated. Put metabolism is involved in their correct maturation [15].

*Ginkgo biloba* L. is a gymnosperm native to China—called a ‘living fossil’—that has significant economic and medicinal value. Due to its high concentration of active constituents such as flavonoids and terpene lactones, it has gained importance for commercial and scientific research purposes in areas such as food, medicine, healthcare, insecticides, ornamental use and ecological conservation [42,43]. Recently, *G. biloba* extract (GBE, containing active ingredients) has been widely in treatments of cerebrovascular and neurological diseases, Alzheimer’s disease, atherosclerosis, and cerebral ischemia/reperfusion (I/R) injury, as well as for anti-inflammatory and antioxidant purposes, along with the suppression of myopia properties due to various diseases [44,45,46]. High levels of active ingredients in Ginkgo require the cultivation of elite species. Research on the technology and theory of SE, genetic transformation, and gene editing are prerequisites for achieving this goal. However, the SE rate of *G. biloba* is extremely low, and lags far behind that of other gymnosperms due to its recalcitrant tissue culture [43]. This restricts the development of Ginkgo in genetic transformation, gene editing and variety improvement. In our previous research, we utilized transcriptomics and cell observations to reveal the mechanism of early embryonic development in *G. biloba* [42]. However, challenges remain in establishing an efficient and stable SE system in *G. biloba*. The mechanisms at each stage of early Ginkgo somatic embryo development remain unclear. The regulation of somatic embryogenesis in Ginkgo is particularly important, especially in the early SE stage.

In this study, we employed bioinformatic methods to analyze the polyamine metabolism-related gene family in *G. biloba*. We examined their chromosomal distribution, cis-regulatory elements, gene structure, distribution of conserved motifs and phylogenetic relationships. Additionally, we investigated the mechanism by which exogenous polyamines (PAs) regulate the early embryonic development of *G. biloba* (conversion from the IC to the EC stage, or the EC to the GE stage), using endosperm as explants. These findings provide a theoretical foundation for understanding the roles of the PAs’ gene families during key stages of early somatic embryogenesis in *G. biloba*. Similarly, this research could help to optimize the somatic embryogenesis system and establish a genetic transformation system in *G. biloba* for elite breeding.

## 2. Results

### 2.1. Identification of PAs Metabolism-Related Gene Families

Using *A. thaliana* protein sequences, a total of 34 genes encoding PAs biosynthesis and metabolism were identified in the Ginkgo genome (https://ginkgo.zju.edu.cn/genome/ftp/, 13 April 2024). Among them, 2 belong to the ADC gene family, 8 to the CuAO gene family, 3 to the SAMDC gene family, 2 to the SPMS gene family, 3 to the SPDS gene family, and 16 to the PAO gene family. The predicted physicochemical properties of the encoded proteins are shown in Table 1.

These genes encode proteins containing between 328 and 1454 amino acids. The molecular weights of these proteins range from 36.04 kDa to 157.78 kDa. The isoelectric points (pI) range from 4.98 to 8.97, with only 3 family members predicted to have pI values above 8.0. Most amino acid pI values fall within the range of 5.0–7.0, indicating that the majority of proteins are acidic. The aliphatic indices range from 74.66 to 99.04. The instability indices range from 28.41 to 51.85; 14 proteins are classified as stable (with an instability index < 40), and 20 as unstable. Additionally, all 34 proteins exhibit negative grand average of hydropathicity (GRAVY) values (Table 1).

### 2.2. Conserved Domains and Structural Analysis of PA-Related Gene Families

**Figure 1:**
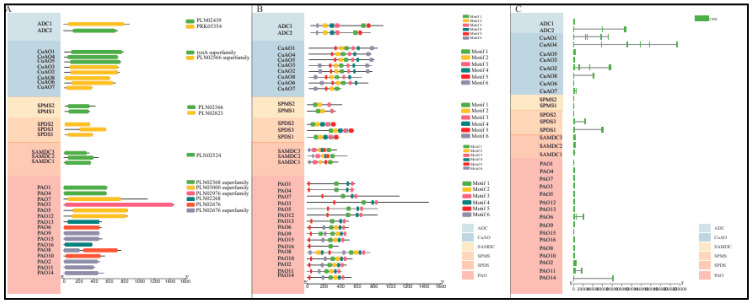
Analysis of conserved structural domains in members of ginkgo polyamine metabolism-related gene family. (**A**). Conserved protein domain architectures among family members. (**B**). Distribution patterns of conserved motifs within the protein sequences. (**C**). Exon–intron organization and genomic arrangements of the corresponding genes.

The conserved motif diagram of the six metabolism-related gene families of PAs in Ginkgo is shown in Figure 1. Despite possessing distinct conserved protein domains, both ADC family members contain six motifs with similar arrangements. Members of the CuAO family are primarily divided into two classes based on their conserved domains, with significant differences in motif composition and arrangement between domain members. Members of the TynA superfamily domain exhibit all six motifs arranged in the order 5–2–1–3–4–6. By contrast, members of the PLN02566 superfamily domain display variations: some contain two copies of motif 6, while others lack motifs 1 and 3. The SPMS and SPDS families can be distinguished by their conserved domains. SPMS members only retain motifs 1, 2 and 3, whereas SPDS members retain all six motifs. A clear correspondence is observed between the motifs and the conserved domains in both families. Of the three SAMDC family members, two retain all six motifs, while one lacks motif 4. However, all three share identical conserved protein domains. The PAO family comprises 16 members and is divided into two major classes. Class I comprises six members that all contain motifs 1, 3, 4 and 5, but exhibit three distinct conserved domains. Class II consists of ten members with three types of conserved domain. Seven of these retain all six motifs, one retains only motifs 1 and 5, PAO13 lacks motifs 2 and 6, and PAO8 uniquely possesses two distinct conserved domains (Figure 1A,B). Gene architecture analysis revealed a diverse exon–intron organization among PA metabolism-related gene families. While some genes are monoexonic, lacking introns, such as ADC1 and CuAO5, others exhibit a complex structure consisting of multiple exons and introns. These genes have a significant variation in total genomic length, for example, ADC2, CuAO4 and PAO14 contain extremely long introns (Figure 1C).

### 2.3. Analysis of Cis-Regulatory Elements in PA-Related Gene Fmilies

**Figure 2:**
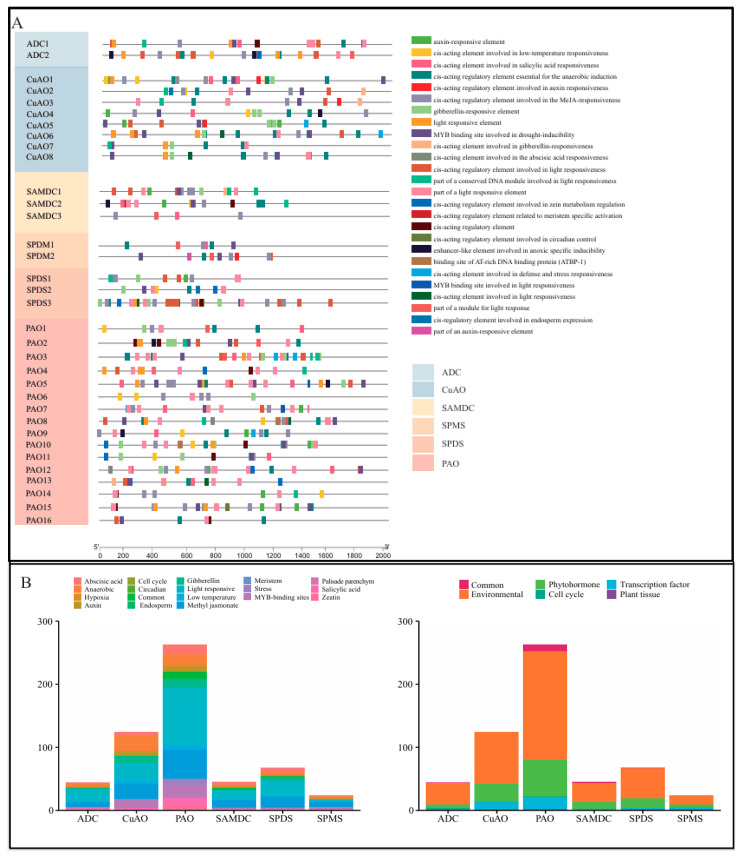
Cis-acting element analysis of a family of genes related to ginkgo polyamine metabolism. *X*-axis: Different gene families (ADC, CuAO, PAO, SAMDC, SPDS, SPMS). *Y*-axis: Frequency of occurrence of cis-elements. (**A**). Distribution of cis-acting elements on different family members; (**B**). Categorical statistics of cis-acting elements by family. **Left**: Detailed functional categories, such as response to abscisic acid, auxin, etc. **Right**: Major functional groups, such as response to environment, phytohormones, etc.

The analysis of the cis-acting elements in the promoters of gene families related to PA biosynthesis and metabolism in *G. biloba* is summarized in Figure 2. A total of 539 cis-acting elements were predicted in the promoters. These elements are primarily associated with responses to phytohormone, abiotic stress, meristem expression, MYB-binding sites, anaerobic induction, cell cycle regulation, etc. The elements of MYB-binding sites related to drought were primarily found in the *GbCuAO* and *GbPAO* families, with 13 and 20 elements, respectively. One to three elements were found in the *GbADC*, *GbSAMDC*, *GbSPDS* and *GbSPMS* families. Furthermore, most of the elements in the promoters are involved in responding to phytohormones, including auxin (AUX), gibberellin (GA), zeatin (ZT) biosynthesis, abscisic acid (ABA), salicylic acid (SA) and methyl jasmonate (MeJA). The number of auxin response elements ranges from two to seven across the six families. The highest numbers of cis-acting elements were found in the responses to GA in the *GbCuAO* family and to ABA and SA in the *GbPAO* family, with 11, 16 and 11 elements, respectively. The largest numbers of elements were found in the response to methyl jasmonate (MeJA), with 8 elements in the *GbADC* family, 24 in the *GbCuAO* family, 12 in the *GbSAMDC* family, 18 in the *GbSPDS* family, 8 in the *GbSPMS* family and 46 in the *GbPAO* family. A significant proportion of these families consist of light-responsive elements. Additionally, elements involved in cell cycle regulation, circadian rhythm regulation, and endosperm expression regulation were found in the *GbCuAO*, *GbSPMS*, and *GbPAO* families. Through the analysis of the promoters of *GbADC2*, *GbSAMDC2*, *GbSPMS1* and *GbPAO* members, some significant differential distribution patterns were observed, shown in Figure 2A,B and Table 2. Most proteins in polyamine-related gene families are associated with plant cell signaling translation, ABA, and environmental stress; in particular, the *GbCuAO*, *GbPAO*, and *GbADC* families are more closely linked to environmental changes (Figure 2B).

### 2.4. Chromosomal Localization and Phylogenetic Tree Analysis of PAs-Related Gene Families

**Figure 3:**
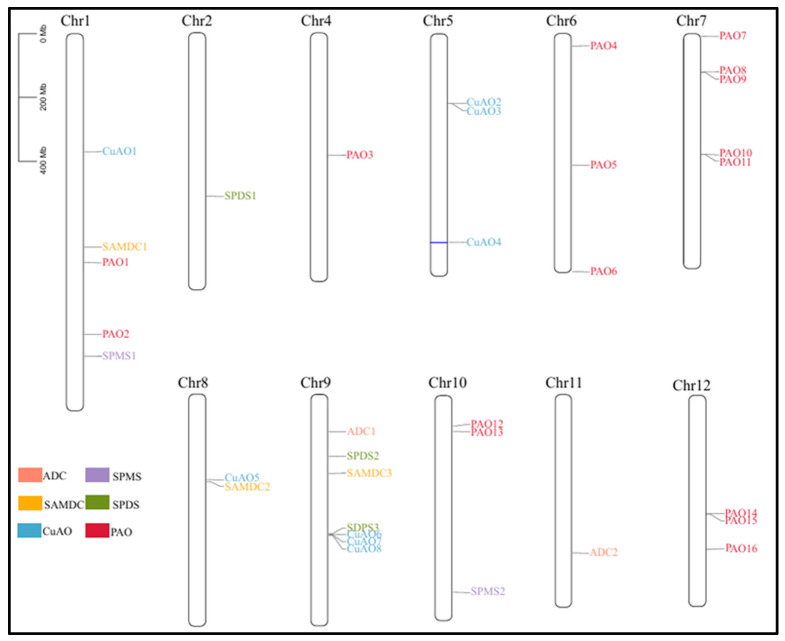
Chromosomal localization analysis of members of each family. Chr: chromosome.

The chromosomal localization of the polyamine metabolism-related gene families in *G. biloba* reveals distinct distribution patterns (Figure 3). The 34 genes are distributed across 11 of the 12 chromosomes, while none are found on chromosome 3. The two ADC family members are localized on chromosomes 9 and 11, and the three SAMDC family members are distributed across chromosomes 1, 8 and 9. The eight CuAO family members are dispersed over five chromosomes: one is found on chromosome 1, one on chromosome 8, and three on both chromosomes 5 and 9. The SPMS family includes one member on chromosome 1 and one on chromosome 10. One SPDS family member is located on chromosome 2, while two reside on chromosome 9. The PAO family spans chromosomes 1, 4, 6, 7, 10 and 12. There are two members on chromosomes 1 and chromosome 10, one on chromosome 4, three on chromosomes 6 and 12, and five on chromosome 7.

### 2.5. Phylogenetic Tree Analysis of PAs-Related Gene Families

**Figure 4:**
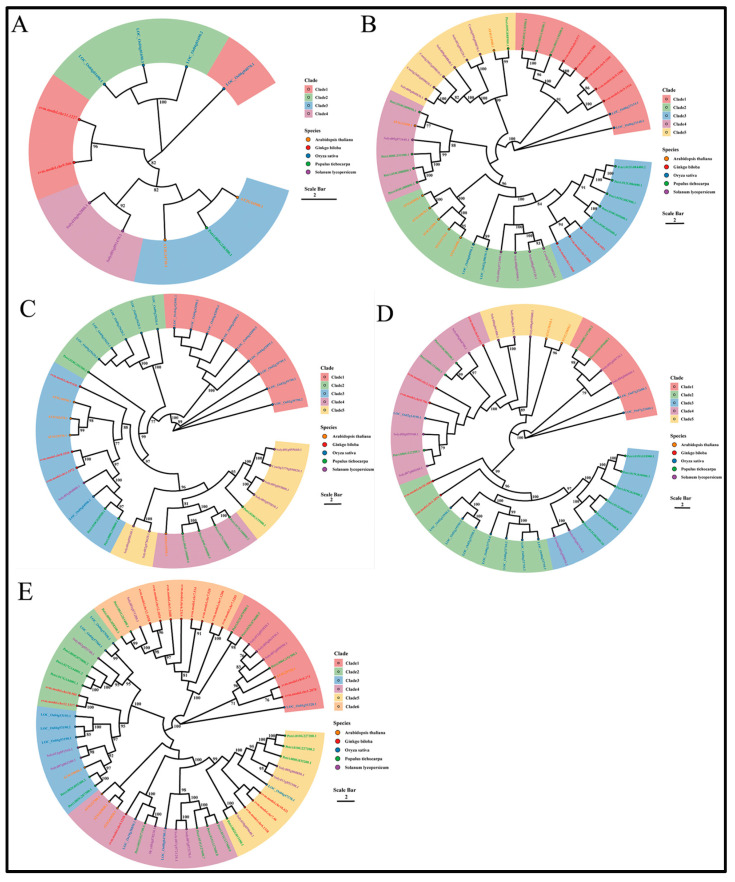
The phylogenetic tree of polyamine metabolism-related gene family members in *G. biloba* and other four species (*Arabidopsis thaliana*, *Populus trichocarpa*, *Solanum lycopersicum* and *Oryza sativmusculus*). (**A**) ADC family evolutionary tree; (**B**) CuAO family evolutionary tree; (**C**) SAMDC family evolutionary tree; (**D**) SPDS and SPMS family evolutionary tree; (**E**) PAO family evolutionary tree. Red font for genes indicates members of the family *Ginkgo biloba.* evm: *Ginkgo biloba*; At (Orange font): *Arabidopsis thaliana*; Potri (Green font): *Populus trichocarpa*; LOC_Os (Purple font): *Oryza sativa*; Solyd, Contig (Blue font): *Solanum lycopersicum*.

Phylogenetic trees were constructed using the maximum-likelihood method based on protein sequence data from *G. biloba*, *Arabidopsis thaliana*, *Populus trichocarpa*, *Solanum lycopersicum* and *Oryza sativa* (Figure 4). The results showed that the ADC proteins from these five species were divided into four main groups: Clade 1, Clade 2, Clade 3, and Clade 4. Clade 1 contained two subgroups, and two of three members were in *G. biloba* ADC proteins. Clades 2, 3 and 4 had three, two, and two members, respectively; these all belong to four other species (Figure 4A).

The phylogenetic tree of CuAO proteins was divided into five groups (Clade 1, Clade 2, Clade 3, Clade 4 and Clade 5). Clades 1 and 3 spanned three plants: *G. biloba*, *P. trichocarpa*, and *O. sativa*. Among these, five members of Clade 1 and three members of Clade 3 belonged to *G. biloba* CuAO proteins. Members of Clades 2, 4 and 5 were found in four other species: *A. thaliana*, *P. trichocarpa*, *S. lycopersicum* and *O. sativa* (Figure 4B).

The SAMDC proteins from the five plants were also classified into five major phylogenetic groups. Of these, Clade 5 contained two subgroups, six of whose seven members belonged to *S. lycopersicum*. Three SAMDC proteins of *G. biloba* were found in Clade 3 (Figure 4C).

The SPDS and SPMS protein sequences were also divided into five major groups based on phylogeny: Clade 1 (5 members), Clade 2 (9 members), Clade 3 (8 members), Clade 4 (10 members) and Clade 5 (5 members). The three SPDS proteins in *G. biloba* (*evm.model.chr9.1497*; *evm.model.chr2.1433*; *evm.model.chr9.796*) were distributed in Clade 4 (Figure 4D). The two SPMS protein sequences (*GbSPMS1* and *GbSPMS2*) were categorized in Clade 2 (*evm.model.chr10.1880*; *evm.model.chr1. 2854*) (Figure 4D).

The PAO protein sequences were separated into six major phylogenetic groups. The 16 PAO proteins in *G. biloba* were distributed across six major groups. Among these, there were eight members in Clade 6, three members in Clade 5, two members in Clade 2 or Clade1, and one member in Clade 4. Non-PAO protein members from *G. biloba* were found in Clade 3 (Figure 4E). The evolution tree of *G. biloba* and other species (*Arabidopsis thaliana*, *Populus trichocarpa*, *Solanum lycopersicum* and *Oryza sativa*) is shown in Figure S1.

### 2.6. Expression Patterns of PAs Family Members in the Somatic Embryogenesis of Ginkgo

**Figure 5:**
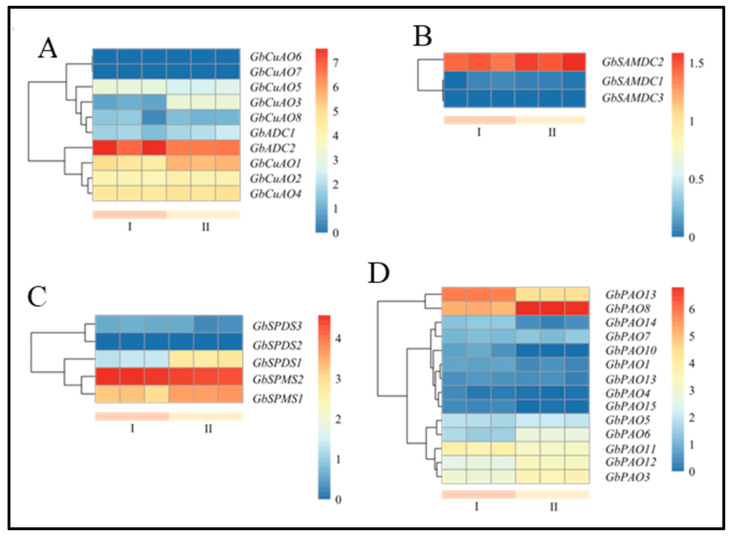
The FPKM of genes involved in polyamine metabolism during endosperm-induced early somatic embryogenesis in *Ginkgo biloba.* (**A**) ADC and CuAO families; (**B**) SAMDC family; (**C**) SPDS and SPMS family; (**D**) PAO family. I: the initial callus (IC stage); II: globular embryos (GE stage).

RNA samples from the initial callus (IC stage) and globular embryo (GE stage) were analyzed by RNA-Seq. The statistics of the sequencing data, volcano plots, GO classification, and KEGG pathway enrichment analysis of DEGs in IC stage vs. GE stage are shown in Table S1 and Figures S2–S4. The two stage-specific gene expression patterns of the PAs’ metabolism-related gene families are shown in Figure 5 (I: IC stage; II: GE stage). The expression levels of *GbADC1* remained consistently low across both developmental stages, while *GbADC2* expression was high during both periods. The FC (fold change) value of *GbADC2* was lower during the GE stage than the IC stage (Figure 5A). *GbCuAO* family members displayed three distinct expression patterns: *GbCuAO6*, *GbCuAO7*, and *GbCuAO8* exhibited low expression in both stages; *GbCuAO1*, *GbCuAO2*, and *GbCuAO4* exhibited high expression in both stages; *GbCuAO1* and *GbCuAO3* were observed to have higher expression in the GE stage than in the IC stage (Figure 5B).

Within the SAMDC family, *GbSAMDC1* and *GbSAMDC3* demonstrated low expression levels in both stages. In contrast, *GbSAMDC2* displayed consistently high expression levels in both stages. Furthermore, *GbSAMDC2* expression was higher in the GE stage than in the IC stage. Within the SPDS family, *GbSPDS2* and *GbSPDS3* exhibited low expression levels in both stages, whereas *GbSPDS1* displayed elevated expression specifically in the GE Stage. *GbSPMS1* and *GbSPMS2* exhibited high expression levels in both stages. Additionally, the FC value of *GbSPMS1* in the GE stage was higher than in the IC stage (Figure 5C).

The expression levels of the *GbPAO3*, *GbPAO8*, *GbPAO6*, *GbPAO12* and *GbPAO13* genes were upregulated in the GE stage, while the expression levels of the *GbPAO4*, *GbPAO10*, *GbPAO14* and *GbPAO15* genes were downregulated compared to the IC stage. No significant difference was observed in the expression levels of the *GbPAO5*, *GbPAO7* and *GbPAO11* genes between the two stages (Figure 5D).

### 2.7. Effect of PAs on the Growth of Ginkgo Endosperm Callus

**Figure 6:**
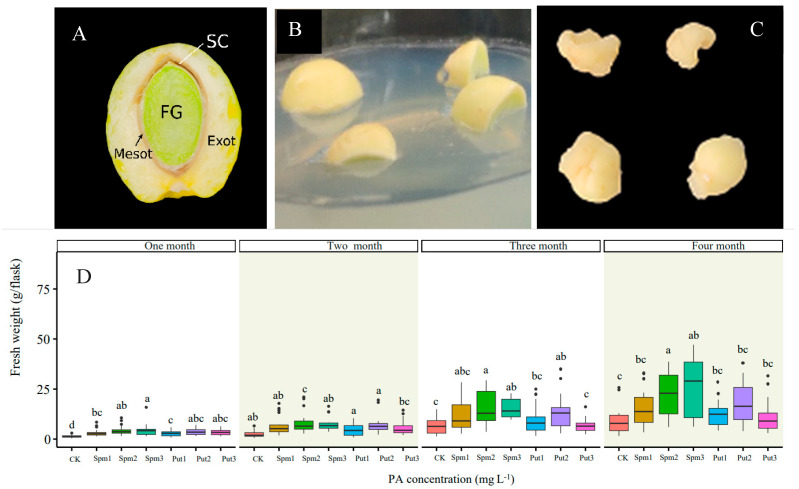
The callus induction of *G. biloba* immature endosperm and changes in fresh weight of callus supplement with different doses of Put and Spm cultured for four months. (**A**) Cross-section of *Ginkgo biloba* fruit at 20–30 July. (**B**) Initial cultivation of immature endosperm. (**C**) Immature endosperm-induced callus. FG: female gametophyte (endosperm); Mesot: middle seed coat; Exot: outer seed coat; SC: seed chamber. (**D**) Fresh weight of callus. Spm1–3: 0.01, 0.1 and 1.0 mg·L^−1^ Spm treatment. Put1–3: 0.01, 0.1, 1.0 mg·L^−1^ Put treatment. CK: control. Data are presented as means ± SD for ten pieces of callus, different lowercase letters indicate significant ANOVA differences (*p* < 0.05). The dots represent outliers.

The 70-day-old callus (Figure 6C) from immature endosperm (Figure 6A,B) was cultured in different PAs treatments for four months. The growth of the endosperm callus is shown in Figure 6D. Ginkgo callus growth was relatively slow during the first two months of culture, with an increase in fresh weight (FW) observed during the third and fourth months. Compared to the control group, the FW of the callus increased significantly under all three Spm treatments from the first to the fourth month of culture. It was also enhanced significantly under the Put1 (0.01 mg·L^−1^) and Put2 (mg·L^−1^) treatments from the second month of culture. During the four-month cultivation period, there was no significant difference in culture FWs between cultures treated with the Put3 (1.0 mg·L^−1^) and the control. This indicated that 0.1–1.0 mg·L^−1^ of Spm and 0.01–0.1 mg·L^−1^ of Put were more effective at promoting endosperm callus growth, whereas 1.0 mg·L^−1^ Put had no significant promoting effect on callus growth (*p* < 0.05). From the third to the fourth cultivation, callus treated with Spm grew faster than that treated with Put.

### 2.8. Histological Observation During Early Somatic Embryogenesis

**Figure 7:**
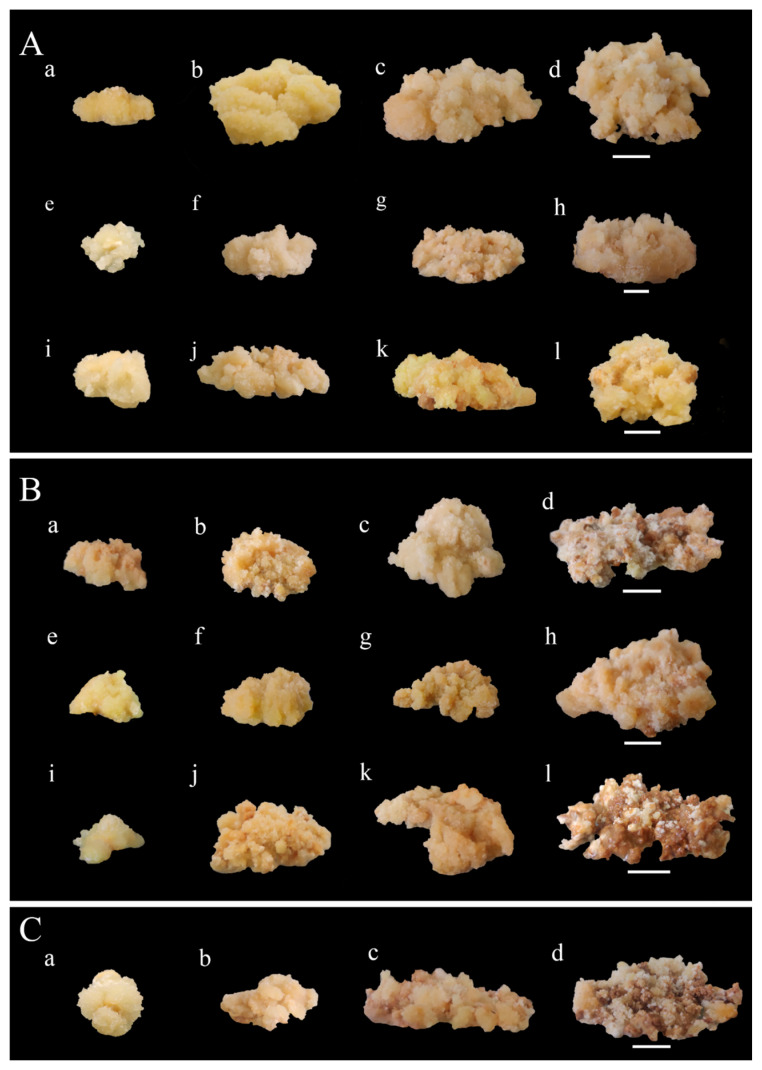
Development processes of Ginkgo endosperm callus after addition of different polyamines. (**A**) Callus status in four months with the addition of Spm; (**B**) callus status in four months with the addition of Put; (**C**) callus status in four months in control. (**a**,**e**,**i**) are for one month of culture, (**b**,**f**,**j**) are for two months of culture, (**c**,**g**,**k**) are for three months of culture, and (**d**,**h**,**i**) are for four months of culture. (**a**–**d**) 0.01 mg·L^−1^ treatment; (**e**–**h**) 0.1 mg·L^−1^ treatment; (**i**–**l**) 1.0 mg·L^−1^ treatment. Scale bars: 1 cm.

**Figure 8:**
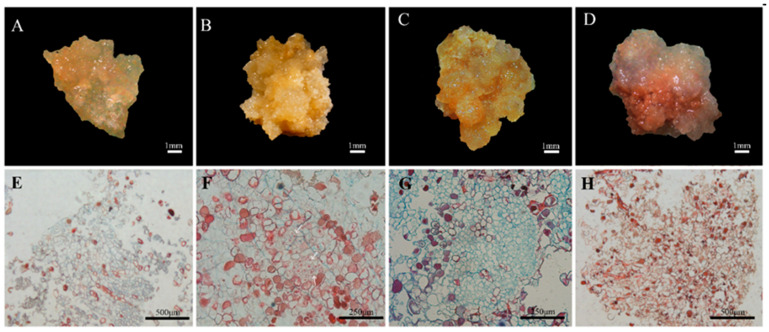
Stereomicroscopic and histological observations of non-embryonic callus (NEC), embryonic callus (EC), and browning callus. (**A**,**E**) are non-embryonic callus cultured in Spm1 and Spm2 (0.01–0.1 mg L^−1^) for 4 months; (**B**,**C**,**F**,**G**) are embryonic callus cultured in 1.0 mg·L^−1^ Spm for 4 months; (**D**,**H**) are browning callus cultured in control for four months.

Ginkgo callus remained in the non-embryogenic callus (NEC) state when treated with Spm1 and Spm2 (0.01–0.1 mg L^−1^) for 4 months of culture (Figure 7A(a–h) and Figure 8A,E). However, embryogenic callus (EC) was observed in cultures treated with 1.0 mg·L^−1^ Spm from the third to fourth month. EC is characterized by organized cell clusters, with a smooth, compact structure, nodular protrusions, and a circular cell ‘growth centre’ (Figure 7A(i–k) and Figure 8B,C,F,G). Embryogenic cell masses were also observed in cultures treated with 0.01 mg·L^−1^ Put from the second month or 0.1 mg·L^−1^ Put from the third month onwards. After four months of culture, the callus surface was densely covered with white, fluffy tissue, displaying a compact structure and a dry surface. This was accompanied by the emergence of globular embryos and localized browning (Figure 7B(b–d,g,h)). However, browning was more severe in the callus treated with 1.0 mg·L^−1^ Put for four months (Figure 7B(l)). The control callus displayed a loose, non-embryogenic structure during the first two months of culture. From the third month onwards, marginal browning and a structural compaction of the callus were observed, which could also initiate the transition to embryogenic callus. However, only a small portion cultures could develop into the EC state; most of the cultures turned brown and died (Figure 7C and Figure 8D,H).

**Figure 9:**
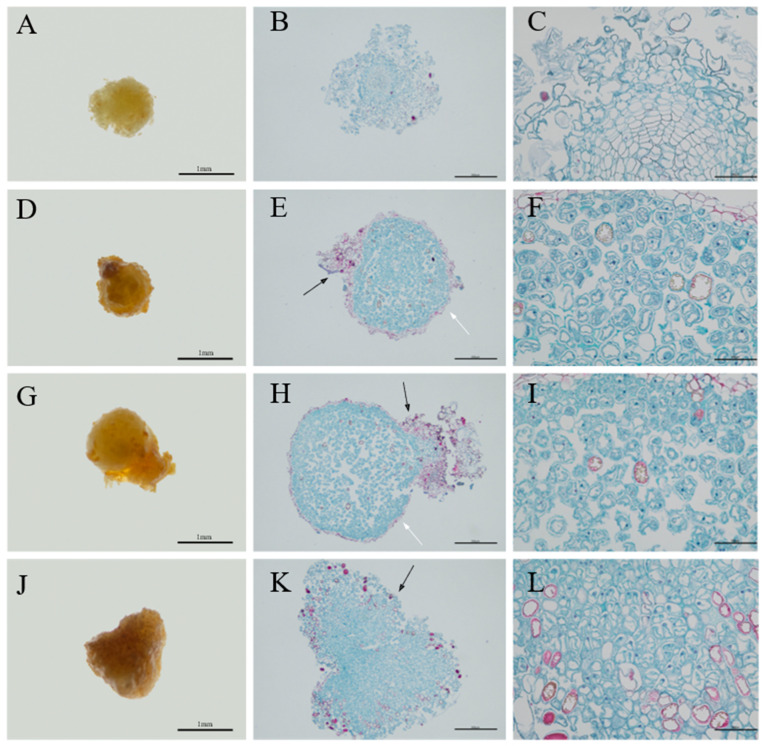
The dynamic morphological development process of the early somatic embryogenesis of Ginkgo through histological observations during initial callus treated with Put1 (0.01 mg·L^−1^) for four months. (**A**–**C**) Embryogenic callus; (**D**–**I**) globular embryo; (**J**–**L**) torpedo embryo. The arrow indicates the tip of the somatic embryonic root. Scale bars: (**A**,**D**,**G**,**J**) 1 mm; (**B**,**E**,**H**,**K**) 500 μm; (**C**,**F**,**I**,**L**) 100 μm.

Histological observations at defined developmental stages of the early somatic embryogenesis of Ginkgo under Put1 (0.01 mg·L^−1^) treatment are shown in Figure 9. During the second month of culture, the callus exhibited EC states with low cytoplasm and dedifferentiated cell masses (Figure 9A–C). In the third month of culture, globular embryos with dense cytoplasm, prominent nuclei, and unequal cell division were observed. The thick-walled cells at the center of the globular embryo constituted the ‘differentiation center’ (Figure 9D–F). In the fourth month of culture, a root tip was observed to be protruding from the globular embryo (Figure 9G–I). Distinct bipolarity with a root tip (black arrow) and a shoot tip (white arrow) appeared (Figure 9H,K) in the globular embryo, and an extended root was shown in the torpedo embryo (TE) (Figure 9K). The cells of the globular embryo were rich in starch grains (Figure 9F,I,L).

### 2.9. Effect of PAs on Antioxidant Enzyme Activities

**Figure 10:**
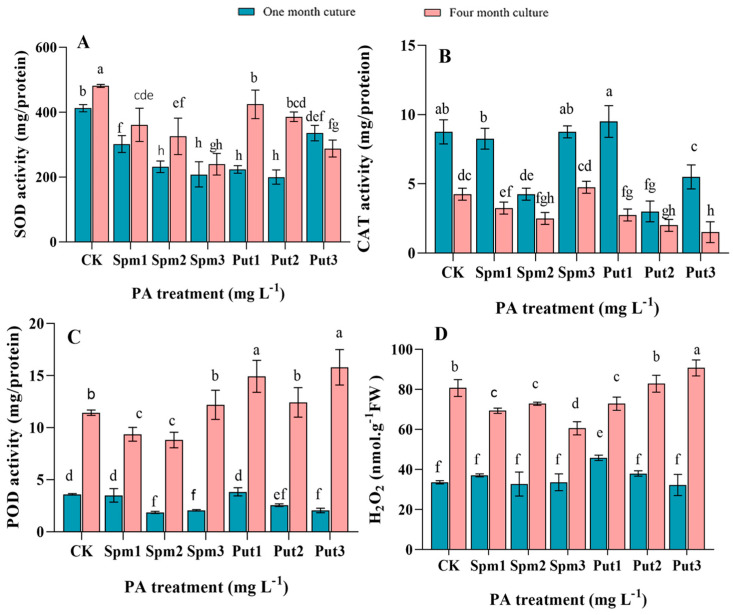
Effects of exogenous PAs on three antioxidant enzyme activities and H_2_O_2_ content in *G. biloba* callus after treatment for one or four months in culture. (**A**) SOD; (**B**) CAT; (**C**) POD; (**D**) H_2_O_2_. Spm1–3: 0.01, 0.1 and 1.0 mg·L^−1^ Spm treatment. Put1–3: 0.01, 0.1, 1.0 mg·L^−1^ Put treatment. SOD: superoxide dismutase; CAT: catalase; POD: Peroxidase; H_2_O_2_: hydrogen peroxide. Data are presented as means ± SD for three independent experiments; different lowercase letters indicate significant ANOVA differences (*p* < 0.05).

The enzyme activities of SOD and POD in each group treated with PAs were higher after four months of culture than after one month, especially with regard to POD activity. Compared to one month of culturing, higher levels of POD were observed under Put1 and Put3 treatments in four months of culture, increased by 289.5% and 673.4%, respectively (Figure 10C) (*p* < 0.05). Similarly, higher levels of SOD were observed under Spm1–2 and Put1–2 treatments, for example, increased by 28.9% and 47.4% under Spm2 and Put1 treatments, respectively (*p* < 0.05) (Figure 10A). Conversely, CAT activity was significantly suppressed in four-month-old cultures under Put1–3 and Spm1–3 treatments compared to one-month-old cultures, for example, decreased by 69.6%, 45.7% and 71.1% in Spm1, Spm3 and Put1 treatments, respectively (*p* < 0.05) (Figure 10B). Higher H_2_O_2_ content was also found in four-month-old cultures under Put and Spm treatments. Higher levels of H_2_O_2_ were observed in CK, Put2 and Put3 treatments, and lower levels in Spm1–3 and Put1 treatments in four-month-old cultures, but still higher by 181.1% in Put3 and 59.1% in Put1 than in one–month–old cultures, respectively (*p* < 0.05) (Figure 10D).

### 2.10. Effect of PAs on Endogenous Hormones

**Figure 11:**
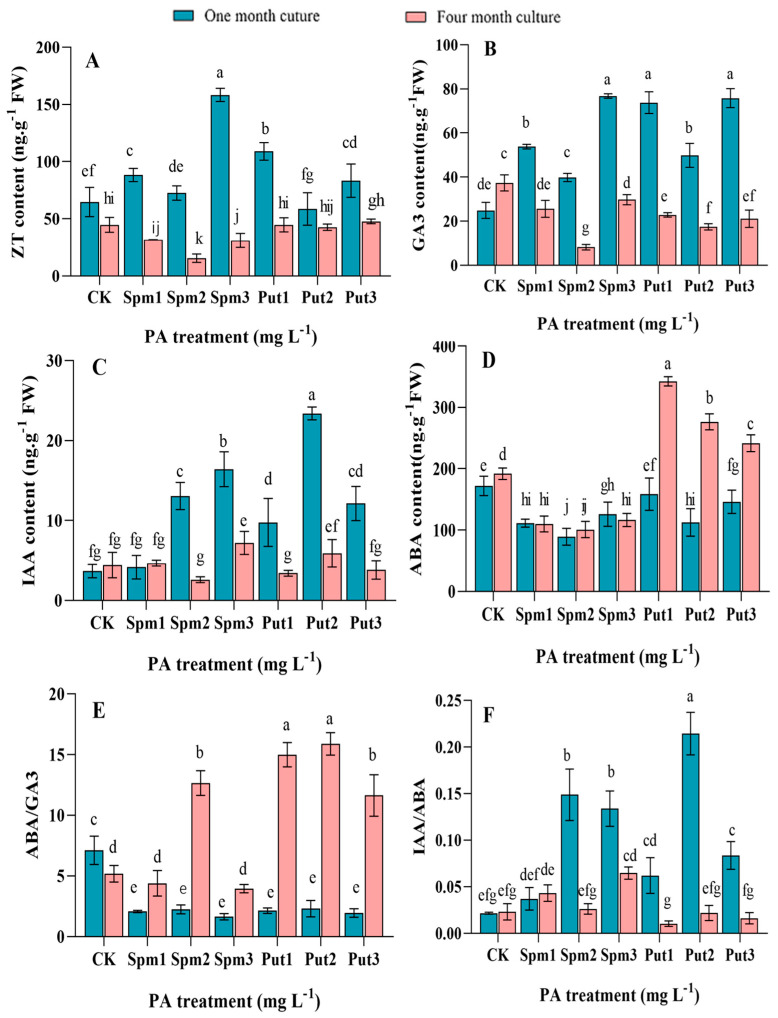
Effects of exogenous PAs on four hormones levels in *G. biloba* callus after treatment in one- or four-month culture. (**A**) ZT; (**B**) GA3; (**C**) IAA; (**D**) ABA; (**E**) ABA/GA3; (**F**) IAA/ABA. Spm1–3: 0.01, 0.1 and 1.0 mg·L^−1^ Spm treatment. Put1–3: 0.01, 0.1, 1.0 mg·L^−1^ Put treatment. IAA: auxin indole-3-acetic acid; GA: gibberellin; ZT: zeatin; ABA: abscisic acid. Data are presented as means ± SD for three independent experiments; different lowercase letters indicate significant ANOVA differences (*p* < 0.05).

The ZT content after four months of culture was significantly lower than after one month of culture under Put and Spm treatments. The same tendency of variation was observed in the contents of GA3 and IAA (indole-3-acetic acid). For example, the levels in Spm3 and Put1 treatments were decreased by 80.3% and 58.9% in ZT, by 158.1% and 226.7% in GA3, and by 56.1% and 65.2% in IAA compared to one month of culture, respectively (*p* < 0.05). Furthermore, after one month of culture, the ZT and GA3 contents in the Spm3, Put1 and Put3 treatments were higher than in the other Spm or Put treatments (Figure 11A–C). Unlike the first three hormones, the ABA content was significantly higher after four months than after one month. The ABA content was lower in the three Spm treatments than in the control treatment under four months of culture. Conversely, the values of ABA in the three Put treatments were higher than in the three Spm treatments and the control treatment. The highest value was found in the Put1 treatment, increased by 78.6% compared to the control in four-month cultures (*p* < 0.05) (Figure 11D). The ABA/GA3 ratio was significantly increased in four-month-old cultures compared to one-month-old cultures, especially in the Spm2 and Put1–3 treatments—increased by 464.8%, 601.1%, 589.0% and 499.5%, respectively (*p* < 0.05) (Figure 11E). Conversely, the IAA/ABA ratio was lower in four-month-old cultures than in one-month-old cultures (Figure 11F).

### 2.11. Expression Levels of Family Member Genes Under Two PAs Treatments

**Figure 12:**
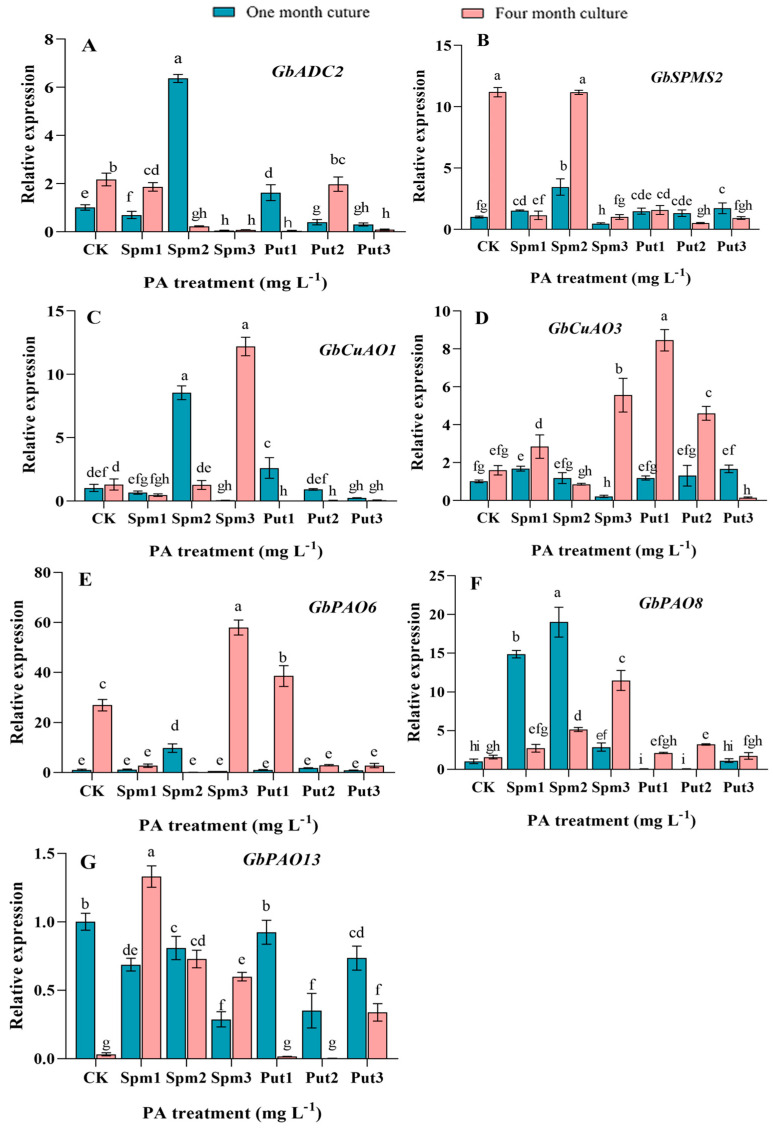
Expression levels of genes related to polyamine metabolism during early somatic embryogenesis in *G. biloba.* (**A**) *GbADC2*; (**B**) *GbSPMS2;* (**C**) *GbCuAO1*; (**D**) *GbCuAO3*; (**E**) *GbPAO6*; (**F**) *GbPAO8*; (**G**) *GbPAO13.* Spm1–3: 0.01, 0.1 and 1.0 mg·L^−1^ Spm treatment. Put1–3: 0.01, 0.1, 1.0 mg·L^−1^ Put treatment. Data are presented as means ± SD for three independent experiments; different lowercase letters indicate significant ANOVA differences (*p* < 0.05).

We selected eight genes with higher expression in the RNAseq analysis for qRT-PCR verification, including *GbADC2*, *GbCuAO1*, *GbCuAO3*, *GbSPDMS2*, *GbPAO6*, *GbPAO8* and *GbPAO13*. The differential expression of seven genes in the callus at one-month-old (Stage I) and four-month-old (Stage II) culture stages are shown in Figure 12.

Compared to Stage I, *GbADC2* was activated in Stage II under Spm1 and Put2 treatment, upregulated by 4.0-fold and 1.7-fold, respectively (*p* < 0.05), but suppressed under Spm2 and Put1 treatment, downregulated by 0.97-fold and 0.98-fold, respectively (*p* < 0.05). The highest expression in Stage I was observed under Spm2 treatment (Figure 12A).

*GbSPMS2* expression was highest in the control and Spm2-treated samples in Stage II. Gene expression was upregulated by 2.2-fold and 1.1-fold under Spm2 and Spm3 treatment, and downregulated by 0.3-fold and 0.5-fold under Put2 and Put3 treatment in Stage II compared to Stage I, respectively (*p* < 0.05) (Figure 12B).

The highest levels of *GbCuAO1* gene expression were found during Stage I under Spm2 treatment and during Stage II under Spm3 treatment. The *GbCuAO1* expression levels in Stage II were increased by 2.3-fold in Spm3 treatment and decreased by 85.3% in Spm2, and by 0.99-fold in Put1 and by 0.98-fold in Put2 compared to Stage I (*p* < 0.05) (Figure 12C).

Compared to Stage I, the *GbCuAO3* exhibited significant upregulation in Stage II under all treatments except Spm2 and Put3, especially under the Spm3, Put1 and Put2 treatments: the expression levels of the *GbCuAO3* in Stage II were 25.1-, 6.2- and 2.5-fold higher than in Stage I, respectively. Compared to the control group, the *GbCuAO3* expression was also found to be upregulated in all treatments except in Spm3 in Stage I and Put 3 in Stage II. Upregulation was particularly high in the Spm3, Put1 and Put2 treatments, at 2.5-, 4.3- and 1.9-fold, respectively, in Stage II compared to the control group (*p* < 0.05) (Figure 12D).

The expression of the *GbPAO6* gene was significantly upregulated in Stage II under Spm3 and Put1 treatment, compared to the control and Stage I. Expression levels in Stage II were 1.15- and 0.43-fold higher under Spm3 and Put1 treatment, respectively, than in the control in Stage I. However, the gene expression was significantly downregulated in Stage I under Spm2 treatment (by more than 8.5-fold) compared to the control (Figure 12E).

The *GbPAO8* gene expression showed upregulation in Stage II under Spm3 and Put1–3 treatments compared to Stage I. For example, the expression levels were 3.0-fold higher in Stage II than in Stage I under Spm3 treatment (*p* < 0.05). Conversely, gene expression was significantly downregulated in Stage II under Spm1 and Spm3 treatment compared to Stage I (Figure 12F).

For *GbPAO13*, the gene expression levels in Stage II were significantly higher (0.94-fold in Spm1, 1.5-fold in Spm3) than those in Stage I (*p* < 0.05). However, the opposite was found for the three Put treatments: *GbPAO13* expression levels were significantly lower in Stage II than in Stage I. Compared to the control, *GbPAO13* gene expression levels were downregulated in all Spm and Put treatments, except Put1 in Stage I. In Stage II, the gene expression was upregulated in all treatments, except Put in 0.01 and 0.1 mg·L^−1^ treatments, compared to the control (Figure 12G).

## 3. Discussion

### 3.1. Structural Characterization of PAs Biosynthesis Genes in Ginkgo

PAs are involved in responding to multiple biological processes such as somatic embryogenesis and environmental stresses [47,48]. Plant cells maintain a state of PA homeostasis and govern cellular development and stress responses through the continuous regulation of synthesis, degradation, and transport [14,47]. Enzymes such as ADC, ODC, SAMDC, SPDS and SPMS are involved in the synthesis of PAs in plants, while CuAO and PAO constitute the PAs catabolism [48]. Unfortunately, the identification of these genes and their structure, as well as the analysis of their transcriptional changes, remains largely unknown in Ginkgo.

In our work, which was based on the third-generation genome data from Arabidopsis, we identified a total of 34 genes involved in PAs biosynthesis and metabolism. These genes were divided into six gene families: ADC, SAMDC, SPDS, SPMS, CuAO and PAO. Most of these genes belonged to the CuAO and PAO gene families, with 8 and 16 members, respectively. The number of polyamine-related genes varied between different plant species. A total of 20 genes involved in PA biosynthesis were also identified in Longyan [12], and 30 genes in wheat [49] were discovered to be involved in PA biosynthesis. However, only ADC, ODC, SPDS, SPMS, and ACL5 were identified in tomato, citrus, and rice [50,51]. Furthermore, ODC was absent from the genomes of *Arabidopsis thaliana* and *Spirodela polyrhiza* [52,53], as well as Ginkgo.

This work reveals that GbCuAO family members contain four to six motifs, the GbADC family contains six motifs, and *GbPAO13* lacks motifs two and six. Interestingly, *GbPAO8* possesses two distinct conserved domains unique to Ginkgo. However, Polyamines-related family members had one to ten motifs in Longyan and tomato [12,50]. The current identification of PAs biosynthesis genes in Ginkgo revealed that they are distributed across 11 of the 12 chromosomes. Some gene families showed an uneven distribution, for example, the ADC family is localized to chromosomes 9 and 11, while CuAO family members are dispersed across five chromosomes. This uneven distribution has also been observed in wheat, Longyan, and tomato [12,49,50].

A total of 539 cis-acting elements were predicted in the promoter region of the polyamine metabolism gene in Ginkgo, mainly associated with responses to light and hormones, as well as with MYB-binding sites. For example, 33 elements were obtained in ABA, 116 in MJ, 175 in response to light, and 41 elements in MYB-binding sites. These mainly belong to the GbCuAO and GbPAO families. The promoters of the polyamine metabolism gene in barley included 260 cis-elements associated with the stress response and 385 elements associated with the growth and development [54]. In all wheat promoters, the identified cis-regulatory elements (CREs) were mainly found in promoter-related responses to light, hormones, the environment and development [49]. These results suggest that polyamines play a role in processes such as light, hormone and stress responses. Studies have demonstrated that adding exogenous polyamines could regulate the expression of genes associated with endogenous polyamine metabolism.

### 3.2. Regulation of PAs in Early Somatic Embryogenesis in Ginkgo

In our preliminary research, we successfully induced the cotyledon stage of somatic embryo in *G. biloba* using immature embryos [42]. In this work, we induced globular-stage (GE) somatic embryos using the immature endosperm of *G. biloba* under PAs treatments. The ECs with a ‘differentiation center’ were found in cultures treated with 1.0 mg·L^−1^ Spm treatment for three to four months, while ECs were found in cultures treated with 0.01–0.1 mg·L^−1^ Put treatment for two or three months, prior to Spm treatment. Furthermore, somatic globular embryos emerged under 0.01–0.1 mmol.L^−1^ Put treatment after four months culturing (Figure 7 and Figure 8). Histological observations revealed that GE cells exhibited distinct bipolarity, with a root tip and a shoot tip (Figure 9). Our results agreed with Longyan’s research on somatic embryogenesis, which indicated that Put and Spm treatment promoted the transformation of EC into GE during the key period of days 9–11 [12]. Our results also aligned with reports which state that 1.0 mM Put and Spm enhance the growth of embryogenic cultures [38], and that polyamines (PAs) promote the conversion of embryogenic calli (EC) into somatic embryos in cotton (*Gossypium hirsutum* L.) [55].

Treatment with Put and Spm promoted the growth of embryogenic cultures of *Araucaria angustifolia.* Synchronously, the levels of endogenous IAA and ABA increased [38]. However, Put and Spm promoted the transformation of EC into GE in Longyan, and led to ABA accumulation and the inhibition of IAA and GA accumulation during this process [12]. In our work, higher levels of ZT, GA and IAA were observed in cultured one-month-old cultures, while higher levels of ABA were evident in four-month-old cultures, particularly under Put1 (0.01 mg·L^−1^) and Spm3 treatments. These changes were particularly remarkable. Changes in endogenous hormone levels were consistent with Spm promoting the EC formation or Put promoting EC-to-GE transformation. Similarly, this was observed in the EC stage, which had a higher ABA content than the NEC stage of *Cucumis melo* [56]. The Spd enhanced the SE efficiency of *Cunninghamia lanceolate*, while the ABA and ZT levels during the SE stage showed an exclusively increasing trend [57]. These results indicated that the IC stage requires higher levels of IAA, GA and ZT, while the EC and GE or CE stages require higher levels of ABA. They also illustrate that ABA might play a major role in the GE morphogenesis.

The literature mostly demonstrated that applications of PAs could stimulate callus organogenesis and somatic embryogenesis. Put induced the generation of new somatic embryos, whereas Spd and Spm resulted in the development and maturation of somatic embryos [3]. High levels of Spd and Spm, and low levels of Put, were found in the embryonic callus of cotton under light treatment. Put levels decreased further while the Spd and Spm levels increased successively during the EC transition into a somatic embryo [58]. Put and Spm levels increased significantly during the early phase of embryo differentiation in cotton, following EC stage transformation. Conversely, Put levels decreased during the somatic embryo stage [55]. Moreover, Put levels were dominant in pre-germination somatic embryos of *Picea rubens* and decreased significantly during somatic embryo development in [26]. The accumulation of Spm was the necessary condition for the transformation of callus into an EC line in both coffee and Longyan plants [12,59]. These findings demonstrate that Put levels were elevated prior to EC formation, whereas a lower endogenous Put content facilitated EC transformation into GE (globular embryos). Changes in PAs levels during somatic embryogenesis might be related to the expression of genes encoding key enzymes involved in polyamine metabolism, as well as to different PA compounds, plant species, and stress factors.

### 3.3. Expression of PAs-Related Genes in the Early Somatic Embryogenesis of Ginkgo

In our work, *GbADC2* gene expression in the callus was upregulated under Spm1 and Put2 treatment, while it was significantly downregulated under Spm2 and Put1 treatment in stage II (four months of culture) compared to Stage I (one month of culture). Gene expression under Spm3 treatment was lower in both stages (Figure 12A). However, *GbSPMS2* gene expression was upregulated under Spm2, Spm3 and Put1 treatments, and downregulated under Put2 and Put3 treatments in Stage II compared to Stage I (Figure 12B). The ADC catalyzes the conversion of arginine to Put, and the SPMS catalyzes the conversion of Spd to Spm. Callus treated with Spm1 and Put2 remained in the NEC state for four months, while the initial callus transformed into the GE stage under Put1 treatment or the EC stage under Spm3 treatment after four months. This indicated that the addition of exogenous Put (such as 0.01 mg L^−1^) could promote the expression of *GbADC2* in Stage I. This led to more Put generation and favoring the initial callus conversion to the EC. The downregulation of this gene’s expression in Stage II indicated that Put content was decreasing, which favored the conversion of the EC stage to the GE stage. Elevated SPMS expression would enhance endogenous Spm levels, which primarily function during somatic embryo development. The expression of the *GbSPMS2* gene was found to be upregulated in Spm-treated cultures in this work. This might favor the synthesis of endogenous Spm and promote the conversion of the IC stage to the EC stage. These results are consistent with the process of cotton SE formation, in which the expression levels of *GhADC1* and *GhADC2* were also increased at the EC stage [55]. The expression of *VvADC* gene was upregulated during the first three weeks, and downregulated after four to five weeks, while the *VvSPMS1* gene expression decreased throughout the culture period of the grapevine somatic embryo aggregates [15]. In addition, the *DlADC2* and *DlSPMS* genes exhibited high expression in the early stages of Longyan SE formation [12]. These results suggest that the ADC and SPMS gene expression is species-specific in plants, and might determine Put and Spm levels in the early phase of embryo differentiation.

Copper ammonia oxidase (CuAO) is responsible for the oxidation of Put in polyamine metabolism. In our work, *GbCuAO1* gene expression was downregulated, and *GbCuAO3* gene expression was upregulated during the transition from the IC (one-month culture) stage to the GE (four-month culture) stage under Put1 and Put2 treatment. Furthermore, a high expression of *GbCuAO1* and *GbCuAO3* was observed following Spm3 treatment after one and four months of culture. These indicated that a significant upregulation of the *GbCuAO3* gene in Stage II might lead to Put degradation. This, in turn, promoted Spm synthesis and enhanced the EC stage conversion to the GE stage. This aligns with the observed changes in the expression levels of *DlCuAOB* and *DlPAO4B*, which were mainly upregulated during the formation of the Longyan SE [12]. A study of the maturation of somatic grapevine (*Vitis vinifera* L.) embryos found that *VvDAO1* expression was significantly downregulated in the second week of culture, and upregulated in the fourth and fifth weeks. Meanwhile, free Put levels were remarkably high in aggregates cultured in the fifth week [15]. These results indicated that upregulating CuAO gene expression favored Put pool balance.

Plant PAOs are important enzymes that play crucial roles in the metabolism of PAs and maintain the balance of the PA pool within the plant. PAOs could be divided into two categories. The first category is known as the ‘PAs terminal catabolism reaction type’, which involves the oxidation and decomposition of Spd and Spm. The second category is known as the ‘PAs back-conversion reaction type’. This class catalyzes PAs back-conversion reactions, i.e., the conversion of spermine (Spm) to spermidine (Spd) and Spd to putrescine (Put) [60]. In our work, *GbPAO6* and *GbPAO8* exhibited higher expression in Spm3 and Put1 treatments, respectively. In contrast, *GbPAO13* exhibited higher expression in the Spm3 treatment, and lower expression in Put1 treatment in Stage II than that in Stage I, which is consistent with the RNA-Seq data. Furthermore, the gene expression levels of *GbPAO6*, *GbPAO8* and *GbPAO13* were also upregulated in the GE stage in our RNA-Seq study. Similarly, the expression of the *VvPAO* gene in the *Vitis vinifera* L. aggregates changed insignificantly when cultured for one week; however, it was significantly upregulated when cultured for four to five weeks, and the free putrescine level increased [15]. In addition, the expression of *GhPAO1* and *GhPAO4* increased dramatically from the NEC to the EC stages in cotton [55]. Hence, the higher expression of the PAO gene supports the second type of PAO, which occurs through back-conversion from Spd to Put. This maintains high levels of free Put during the SE formation stage.

### 3.4. The Mechanism of PAs Promoting the Early Somatic Embryogenesis of Ginkgo

**Figure 13:**
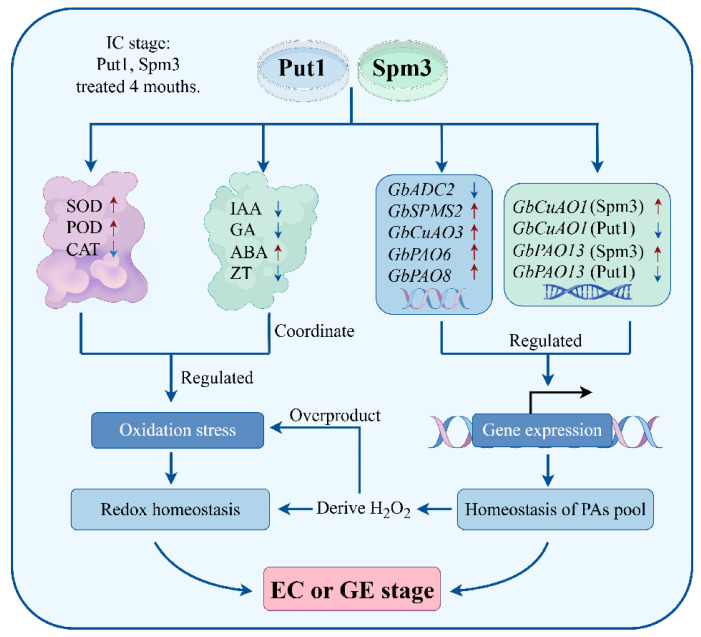
The mechanism by which polyamines induce the early somatic embryogenesis of *G. biloba*. Put1: 0.01 mg·L^−1^ Put treatment; Spm3: 1.0 mg·L^−1^ Spm treatment. IC: initial callus; EC: embryogenic callus; GE: globular embryo. ↑: upregulation; ↓: downregulation.

Recent studies have shown that the idea ‘the more PAs in plant, the better’ does not always hold true [14]. The dynamic changes in PAs amounts are regulated by the free PA pool, their synthesis and degradation, their distribution and transport, and the expression level of genes encoding PA enzymes. PAs homeostasis in plant cells further regulates cell development and various physiological responses such as stress responses [14,47,61]. The courses of CuAO catalyzing the conversion of Put to NH_4_^+^, and PAO catalyzing the conversion of Spd and Spm, could all generate hydrogen peroxide (H_2_O_2_) [3,61]. H_2_O_2_ is one of the ROS (reactive oxygen species) involved in the signaling pathways of PAs, ABA, jasmonic acid (JA), salicylic acid, ethylene and more [5,6,62]. Research has indicated that PAs-derived H_2_O_2_, acting as a byproduct, mediates ABA and NO signaling during stomatal closure, as well as being involved in the antioxidant defense system, cell wall maturation, and stress-induced stiffening [4,5,63]. The *AtPAO5* gene, which catalyzes the conversion of Spm to Spd, was found to exhibit the greatest transcriptional responsiveness to salinity stress, and to be associated with an early increase in ABA biosynthesis [5]. In our work, the high expression of GbCuAOs and GbPAOs suggested that the formation of EC and GE in Ginkgo was accompanied by the degradation of Put or Spm, resulting in the production of H_2_O_2_ and causing oxidative stress. However, antioxidant enzyme activity (SOD and POD) increased and could maintain ROS homeostasis in the plant. Thus, PAOs are important for ROS homeostasis [8]. This may be a beneficial or necessary stage for the formation of somatic embryos. The mechanism by which polyamines induce the early somatic embryogenesis of *G. biloba* is shown in Figure 13.

## 4. Materials and Methods

### 4.1. Plant Materials and Callus Induction

The experiment was performed in Nanjing Forestry University (NJFU) from 2022 to 2025. The test material was callus from the immature endosperm, prepared using Ma’s methods [64]. The immature endosperm was obtained from a 30-year-old *G. biloba* tree on the NJFU campus. Slices of the endosperm were inoculated onto the Murashige and Skoog (MS) medium with the cut side facing down, and cultured for 40 days to induce callus formation. The MS medium was supplemented with 1.5 mg·L^−1^ naphthaleneacetic acid (NAA), 1.0 mg·L^−1^ 6-benzylamine purine (6-BA) and 0.25 mg·L^−1^ kinetin (KT). The cultures were then subcultured for a further 30 days in an MS medium containing 1.0 mg·L^−1^ NAA and 1.0 mg·L^−1^ 6-BA to promote proliferation.

### 4.2. PA Treatment

After filtering and sterilizing different doses of Put and Spm, these were added to an MS medium containing 0.25 mg·L^−1^ NAA, 1.0 mg·L^−1^ 6-BA and 0.2 mg·L^−1^ thidiazuron (TDZ). The doses of the added PAs are as follows: 0.01, 0.1, and 1.0 mg·L^−1^ (final concentration in medium) for Spm (labelled Spm1, Spm2 and Spm3); and 0.01, 0.1, and 1.0 mg·L^−1^ for Put (labelled Put1, Put2 and Put3). The control (CK, no PAs addition) was given the same amount of sterile water. Then, the callus-resulting endosperm inductions were transferred to a medium containing PAs for somatic embryogenesis. Four pieces of callus were inoculated into each bottle; twelve bottles were used per treatment, in triplicate. All the above culture media also contained 30 g·L^−1^ sucrose and 6.5 g·L^−1^ agar. The pH was adjusted to 5.8. All cultures were cultivated for four months in the dark at 25 ± 1 °C, and subcultured once every 30 days.

Samples of callus treated with Put and Spm were collected during culturing from the first to the fourth month. One portion of the collected samples was used to determine the growth parameters and another portion was used for histological observation. The remaining samples were immediately frozen in liquid nitrogen and stored at −80 °C for physiological and molecular biological analysis.

Fresh weight of callus (g) = the total weight after inoculation of callus (Wt) − the weight of the empty bottles and the culture medium (Wi).

The fresh weight was measured on the last day of each month during the callus tissue culture process, for a total of four times.

### 4.3. Morphological Observation

A stereomicroscope was used for cell morphological observation. For histological analyses, callus pieces were fixed in a formaldehyde–acetic acid–ethanol (FAA) solution (formaldehyde–acetic acid–70% ethanol = 5:5:90 (*v*/*v*/*v*)). The fixed tissues were then dehydrated in aqueous solutions of tertiary butyl alcohol and cut into 6 μm-thick sections using a rotary microtome according to Li [65]. All sections were viewed using a Leica DM500 light microscope (Leica, Wetzlar, Germany). 

### 4.4. Identification of PA Metabolism-Related Genes in G. biloba

The genes involved in the biosynthesis and metabolism of polyamines (PAs) in Arabidopsis were obtained from TAIR (v12) (https://www.arabidopsis.org/). These gene numbers were referenced to Guo et al. [66]. The local BLAST function in TBtools (v 2.021) was used to align the base sequences obtained from TAIR [67]. The reliability of gene sequences related to PA biosynthesis identified in the *G. biloba* genome was verified using NCBI’s Batch CD-Search (https://www.ncbi.nlm.nih.gov/Structure/bwrpsb/bwrpsb.cgi/, 13 April 2024) and SMART tools (https://smart.embl.de/smart/change_mode.cgi, 13 April 2024) [40,68]. ExPASy (https://www.expasy.org/) was used to detect the physicochemical properties of polyamine biosynthetic proteins, including determining the number of amino acids (aa), the molecular weight (MW), the isoelectric point (PI), the instability index (II), the aliphatic index (AI) and the hydrophilicity. PlantCARE (https://bioinformatics.psb.ugent.be/webtools/PlantCARE/, 13 April 2024) was used to conduct an analysis of the cis-acting elements in the 2000 bp fragment located upstream from the starting codon of the gene. MEME (https://meme-suite.org/meme/tools/meme, 13 April 2024) was used to analyze protein motifs. Finally, TBtools (v2.021) software was used to visualize the analysis of conserved domains and motifs.

### 4.5. Chromosomal Localization and Phylogenetic Analysis

The visualization function in TBtools (v2.021) was used to identify the locations of genes associated with polyamine metabolism on the chromosome [67]. The polyamine biosynthesis genes in *Arabidopsis thaliana* were obtained from TAIR (v12). The amino acid sequences of *Oryza sativa* L., *Populus trichocarpa* Torr. & Gray, and *Solanum lycopersicum* L. were obtained from Phytozome (v13) (https://phytozome-next.jgi.doe.gov/). The protein sequences were aligned using MAFFT (v7.310) (Multiple Alignment using Fast Fourier Transform). Phylogenetic trees were constructed using IQ-TREE 2 (Iterative Quotient Tree) with the 1000 bootstrap replicates. These trees were then visualized using iTOL [69,70].

### 4.6. RNA-Seq Data Analysis of PAs Biosynthesis Gene Expression

Existing transcriptomic data of callus tissues (with three biological replicates) from the initial callus (IC) stage and globular embryo callus (GE) stage, which were induced by *G. biloba* endosperm, were obtained from our research group. RNA-Seq analysis was performed using an Illumina NovaSeq 6000 (Illumina, Inc. San Diego, USA). After removing the low-quality regions from raw RNA-Seq data, the GC and Q30 contents of the clean data were calculated as quality scores. The stage-specific expression patterns of the PA metabolism-related gene families were analyzed using this transcriptomic data. After performing quality control of the transcriptome, gene expression at the isoform level was estimated using FPKM (fragments per kilobase of transcript per million mapped reads). The R package (version 4.2.2) was then used to visualize the data and generate heatmaps of normalized expression profiles. Due to discrepancies in gene ID between the transcriptomic data and the *G. biloba* genome version used for gene family analysis, BLAST alignment was performed to resolve the mismatches and retrieve the homologous gene ID from the transcriptomic dataset. Differentially expressed genes (DEGs) were calculated using the DESeq2 R package (version 4.2.2) with *p*-value < 0.05 and |log_2_ FC| ≥ 1.0. The assembled unigenes were annotated to NR (NCBI non-redundant protein sequences), GO (Gene Ontology), and KEGG (Kyoto Encyclopedia of Genes and Genomes); the analysis above was performed using BMKCloud (Beijing Biomarker Technologies Co., LTD, Bejing, China) (www.biocloud.net).

### 4.7. Endogenous Hormone and Antioxidase Contents in G. biloba Determination

The levels of indole-3-acetic acid (IAA), gibberellin (GA3), zeatin (ZT), and abscisic acid (ABA) were determined by high-performance liquid chromatography (HPLC), according to the methods described by Chen et al. and Li et al. [71,72]. The reference standards for indole-3-acetic acid, zeatin (purity ≥ 98%), gibberellic acid (purity ≥ 90%), and abscisic acid (purity ≥ 99%) were all purchased from Shanghai Yuanye Bilogical Technology Co., Ltd., Shanghai, China) The fresh calluses were homogenized in liquid nitrogen and extracted using a cold methanol solution (80% (*v*/*v*) containing butylated hydroxytoluene (1 mmol.L^−1^), then centrifuged at 3000× *g* for 15 min. After storage at 4 °C for 24 h, the resulting supernatant was purified through a C-18 Sep-Pak cartridge extraction column (Agilent Technologies Inc., Santa Clara, CA, USA), then the extract was purified with a 0.22 μm filter. The purified extracts were then analyzed using HPLC (Agilent 1260), with chromatography column Eclipse XDB-C18 (4.6 × 150 mm, 5.0 μm) (Agilent Technologies Inc., Santa Clara, CA, USA). The mobile phase was a mixture of methanol and 0.075% acetic acid (45:55) *v*/*v*) at a flow rate of 1.0 mL min^−1^. The column temperature was set to 30 °C and the detection wavelength was set to 254 nm.

The activities of SOD (superoxide dismutase), CAT (catalase), POD (peroxidase) and H_2_O_2_ were determined, as described in references [73,74].

### 4.8. RNA Iisolation and Quantitative Real-Time PCR (qRT-PCR) Analysis

Total RNA was extracted from the PA-treated and untreated calluses using the Total RNA Isolation Kit (Tiangen Biochemical Technology Co., Ltd., Beijing, China) extraction protocol. Following the manufacturer’s instructions for the GenStar cDNA Synthesis Kit (GenStar Biotechnology Co., Ltd., Beijing China) residual genomic DNA was eliminated and first-strand cDNA synthesized for qRT-PCR. The qRT-PCR primers were designed using PrimeQuest (https://sg.idtdna.com/pages/tools/primerquest, 13 April 2024), with *GbGAPDH* referenced as the internal control gene (Table S1). The amplification protocol comprised three steps: initial denaturation step at 95 °C for 2 min, followed by 40 cycles of denaturation at 95 °C for 15 s, and annealing at 60 °C for 15–30 s. After amplification, the fluorescence and melting curves were analyzed to confirm specific amplification. Each reaction was performed in triplicate. Relative gene expression levels involved in PA metabolism were calculated using the 2^(−ΔΔ)^ Ct method [75], with expression levels in the first month of the control treatment set as the reference standard.

### 4.9. Statistical Analysis

Correlation analysis and data visualization were performed in R (version 4.2.2) using the ggplot2 package for plotting. The differences between the treatments were statistically evaluated using a one-way analysis of variance (ANOVA), followed by a Fisher’s least significant difference (LSD) test for multiple comparisons.

## 5. Conclusions

In conclusion, 34 genes associated with polyamine metabolism in the third-generation genome of Ginkgo were identified. The GbADC, GbSAMDC, GbSPMS, GbSPDS, GbCuAO and GbPAO gene families comprise 2, 3, 2, 3, 8, and 16 members, respectively. The 539 cis-acting elements mainly respond to phytohormones, abiotic stress and meristem expression, as well as to MYB-binding sites. The families are distributed across 11 of the 12 chromosomes.

Embryogenic callus (EC) or globular embryos (GEs) were observed in cultures treated with 1.0 mg·L^−1^ Spm (Spm3) or 0.01 mg·L^−1^ Put (Put1) for four months. Higher levels of ABA, SOD and POD activity and H_2_O_2_, and lower levels of IAA, GA and ZT, were found in the four-month-old cultures, indicating that the EC or GE stage might require SOD and POD balancing the H_2_O_2_ produced by the oxidation of polyamines, and this stage needs a high level of ABA.

The gene expressions of *GbADC2*, *GbSAMDC2*, *GbSPMS1*, *GbCuAO1* and *3*, and *GbPAO3*, *8*, *6* and *13* in *G. biloba* were upregulated in the GE stage compared to the IC stage using RNA-seq analysis. qRT-PCR verified *GbSPMS2*, *GbCuAO3*, *GbPAO6*, and *GbPAO8* were also upregulated, while *GbADC2* was downregulated in four-month-old cultures under Put1 or Spm3 treatment. Additionally, *GbCuAO1* and *13* gene expressions were upregulated when treated with Spm3, while the two genes were downregulated when treated with Put3 for four months.

These results indicated that appropriate polyamine treatment could promote the conversion of ICs to ECs or GEs in *G. biloba* callus. During this process, the expressions of genes related to PAs synthesis and decomposition were enhanced, thereby maintaining the polyamine’s pool homeostasis in the cell. At the same time, the increased antioxidant enzyme activities and ABA levels helped to maintain the homeostasis of the H_2_O_2_ in *G. biloba*.

## Figures and Tables

**Table 1:** Properties of the proteins involved in PA biosynthesis and metabolism genes.

Gene Name	Gene ID	Protein Length (AA)	MW (kDa)	pI	ID	AI	GRAVY
*GbADC1*	evm.model.chr11.1227	715	77.56	5.57	44.23	89.71	−0.03
*GbADC2*	evm.model.chr9.566	866	98.07	6.40	46.12	87.11	−0.293
*GbCuAO1*	evm.model.chr1.1060	787	88.24	6.42	42.73	77.79	−0.372
*GbCuAO2*	evm.model.chr5.1800	714	80.42	6.49	45.18	74.66	−0.402
*GbCuAO3*	evm.model.chr8.1021	749	83.75	6.21	41.64	78.49	−0.316
*GbCuAO4*	evm.model.chr5.580	722	81.15	5.99	34.42	89.25	−0.226
*GbCuAO5*	evm.model.chr5.577	729	82.19	6.31	34.59	82.65	−0.327
*GbCuAO6*	evm.model.chr9.1510	606	68.24	5.97	32.95	82.49	−0.239
*GbCuAO7*	evm.model.chr9.1508	679	75.14	8.17	33.04	77.23	−0.291
*GbCuAO8*	evm.model.chr9.1509	365	41.05	5.96	43.62	88.38	−0.084
*GbSAMDC1*	evm.model.chr9.930	339	38.16	5.2	47.75	77.08	−0.158
*GbSAMDC2*	evm.model.chr8.1036	462	51.08	5.18	51.85	74.74	−0.25
*GbSAMDC3*	evm.model.chr1.1972	358	39.99	5.77	42.06	84.25	−0.052
*GbSPMS1*	evm.model.chr10.1880	403	44.31	5.14	48.89	84.59	−0.141
*GbSPMS2*	evm.model.chr1.2854	328	36.04	5.76	45.78	76.4	−0.234
*GbSPDS1*	evm.model.chr9.796	333	37.30	5.57	29.06	90.39	−0.162
*GbSPDS2*	evm.model.chr9.1497	545	60.63	6.81	40.78	77.27	−0.396
*GbSPDS3*	evm.model.chr2.1433	369	40.87	6.06	28.41	77.4	−0.351
*GbPAO1*	evm.model.chr1.2078	573	62.63	5.79	33.65	84.07	−0.259
*GbPAO2*	evm.model.chr6.171	563	62.32	5.36	37.28	86.54	−0.376
*GbPAO3*	evm.model.chr7.46	1104	122.61	6.39	41.53	91.16	−0.057
*GbPAO4*	evm.model.chr4.1258	1454	157.78	5.23	43.49	80.99	−0.428
*GbPAO5*	evm.model.chr6.1338	842	92.22	8.97	45.96	89.67	−0.231
*GbPAO6*	evm.model.chr10.421	840	92.10	6.98	49.39	87.67	−0.244
*GbPAO7*	evm.model.chr10.505	498	55.33	5.87	41.28	94.18	−0.043
*GbPAO8*	evm.model.chr6.2315	495	55.74	6.35	38.55	80.75	−0.227
*GbPAO9*	evm.model.chr7.520	468	52.90	5.65	44.16	87.91	−0.121
*GbPAO10*	evm.model.chr12.1039	507	56.63	4.98	36.13	80.77	−0.337
*GbPAO11*	evm.model.chr12.1317	376	41.92	8.39	40.47	99.04	−0.013
*GbPAO12*	evm.model.chr7.514	752	85.41	5.09	35.96	83.86	−0.282
*GbPAO13*	evm.model.chr7.1205	540	61.72	6.13	41.58	80.85	−0.323
*GbPAO14*	evm.model.chr1.2688	472	52.99	6.04	32.09	84.26	−0.345
*GbPAO15*	evm.model.chr7.1206	412	46.81	5.29	39.59	76.14	−0.238
*GbPAO16*	evm.model.chr12.1038	527	59.11	5.3	38.54	78.44	−0.37

MW: molecular weight; AA: amino acid; ID: instability index; AI: aliphatic index; GRAVY: grand average of hydropathicity.

**Table 2:** Categorical statistics of cis-acting elements by family.

Response	*GbADC*	*GbCuAO*	*GbSAMDC*	*GbSPDS*	*GbSPMS*	*GbPAO*	Total
MYB-binding sites	1	13	2	2	3	20	41
Auxin response	3	7	2	2	2	7	23
GA	-	9	2	5	-	2	20
ABA	3	-	5	7	2	16	33
ABA–GA crosstalk	-	-	-	-	-	15	15
ZT	2	-	-	2	-	9	13
SA	1	3	2	-	1	11	18
MJ	8	24	12	18	8	46	116
Anaerobic induction	3	23	2	-	3	17	48
Light	20	24	14	24	4	89	175
Low temperature	1	5	1	1	-	9	25
Hypoxia induction	2	-	1	-	-	3	6
General regulatory	1	-	-	-	-	-	1
Cell cycle regulation	1	-	-	-	1	1	3
Circadian rhythm regulation	-	-	-	-	-	1	1
Endosperm expression regulation	-	-	-	1	-	-	1
Total	39	79	32	46	17	194	539

MYB-binding sites: light-responsive and drought-related MYB-binding sites; GA: gibberellin response; ABA: abscisic acid response; ZT: zeatin response; SA: salicylic acid response; MJ: methyl jasmonate response. -: the gene had no response in the metabolism.

## Data Availability

All data generated or analyzed during this study are included in the article and its Supplementary Materials.

## Supplementary Materials

The following supporting information can be downloaded at: https://www.mdpi.com/article/10.3390/plants15111617/s1: Figure S1. The evolution tree between *G. biloba* and *Arabidopsis thaliana*, *Populus trichocarpa*, *Solanum lycopersicum* and *Oryza sativmusculus*. Figure S2. Volcanic diagrams showing the number of DEGs (differential genes) in IC stage (initial callus) vs. GE stage (globular embryo); Figure S3. GO classification of DEGs by transcriptome analysis; Figure S4. KEGG pathway enrichment analysis of DEGs in IC stage vs. GE stage; Table S1. Real-time quantitative PCR primer.

## Abbreviations

The following abbreviations are used in this manuscript:

SE	Somatic embryogenesis
IC	Initial callus
EC	Embryogenic callus
GE	Globular embryo
Put	Putrescine
Spm	Spermine
