# Supplementary material for: Polyamine-Related Gene Families Identification and Regulatory Effects on Early Somatic Embryogenesis via Modulating Gene Expressions and Hormone Levels in Ginkgo biloba
Source: Plants (Basel). 2026 May 25;15(11):1617. doi: 10.3390/plants15111617 (PMC13258931; doi:10.3390/plants15111617)
Supplement: Supplementary file 1 [file plants-15-01617-s001.zip › Supplemenary FIGURE.pdf]

Polyamine-Related Gene Families Identification and Regulatory Effects on Early Somatic Embryogenesis via Modulating Gene Expressions and Hormone Levels in *Ginkgo biloba*

Supplementary Figures

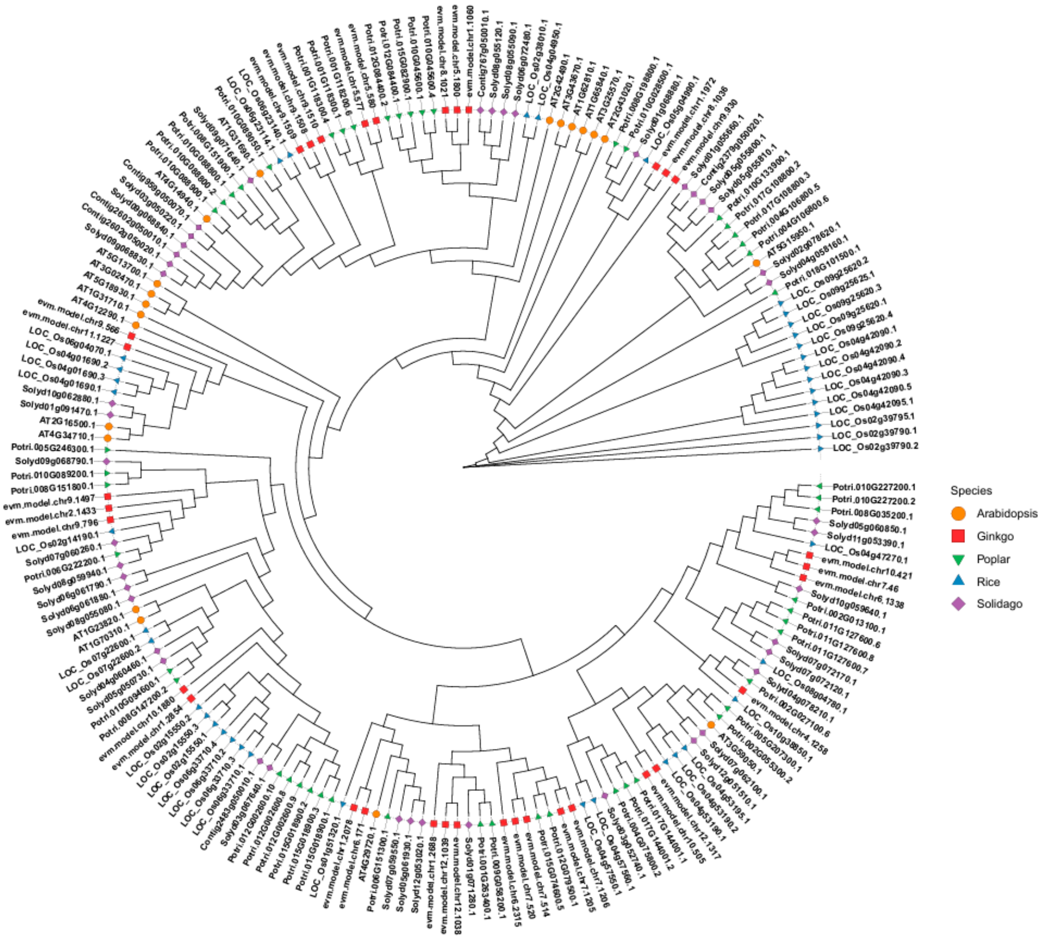

Figure S1. The evolution tree of between *G. biloba* and *Arabidopsis thaliana*, *Populus trichocarpa*, *Solanum lycopersicum* and *Oryza sativmusculus.* evm: Ginkgo biloba; At: Arabidopsis thaliana; Potri: *Populus trichocarpa*; LOC_Os: *Oryza sativa*; Solyd: *Solanum lycopersicum*.

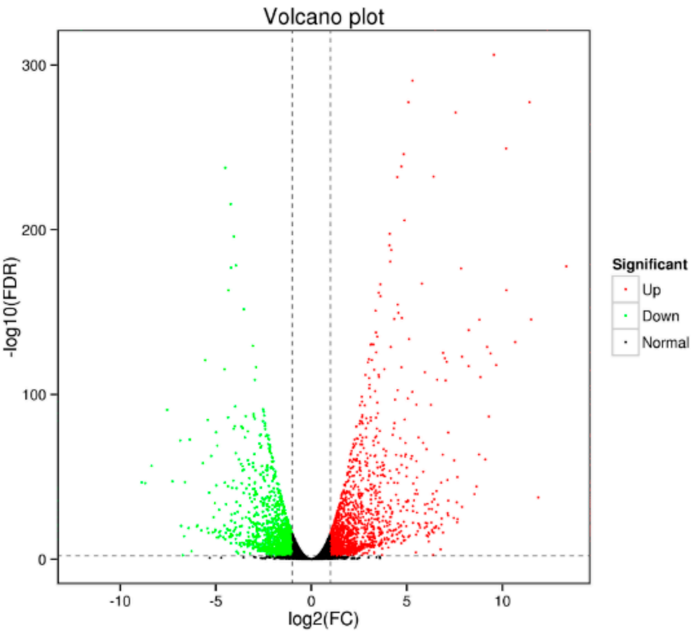

Figure S2. Volcanic diagrams showing the number of DEGs (differential genes) in IC stage (initial callus) vs GE stage (globular embryo). Red dots denote significantly upregulated genes, whereas green dots denote significantly downregulated genes.

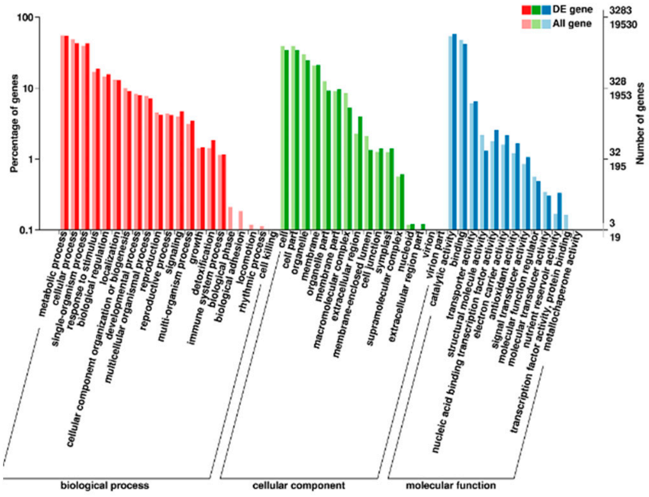

Figure S3. GO classification of DEGs by transcriptome analysis. The three main GO categories (biological process, cellular component and molecular function) are summarized. The x-axis indicates Go-terms, and the y-axis indicates the number of DEGs in IC stage vs GE stage.

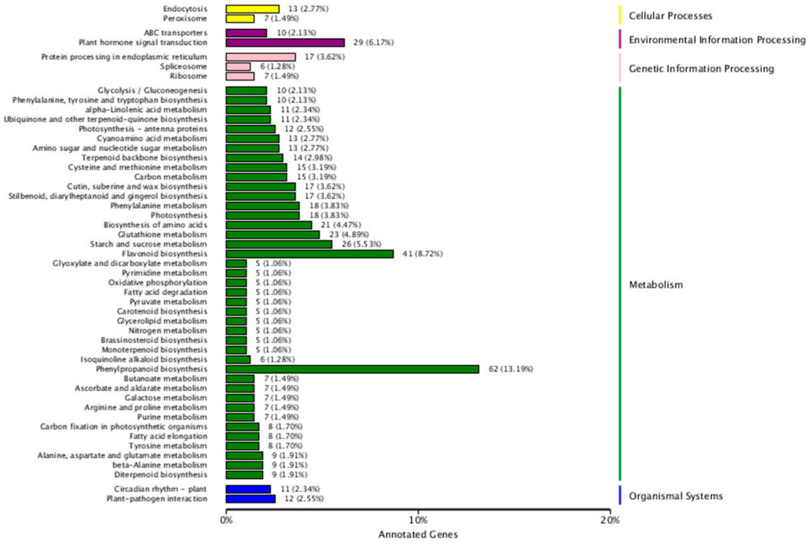

Figure S4. KEGG pathway enrichment analysis of DEGs in IC stage vs GE stage. The pathway name is indicated by the vertical coordinate, and the percent of annotated genes is indicated by the horizontal coordinate.
