# Supplementary material for: Polyamine-Related Gene Families Identification and Regulatory Effects on Early Somatic Embryogenesis via Modulating Gene Expressions and Hormone Levels in Ginkgo biloba
Source: Plants (Basel). 2026 May 25;15(11):1617. doi: 10.3390/plants15111617 (PMC13258931; doi:10.3390/plants15111617)
Supplement: Supplementary file 1 [file plants-15-01617-s001.zip › Supplement TABLE.pdf]

Polyamine-Related Gene Families Identification and Regulatory Effects on Early Somatic Embryogenesis via Modulating Gene Expressions and Hormone Levels in *Ginkgo biloba*

Tables

Table S1 Real-time quantitative PCR primer

Gene	Forward peimer（5'-3'）	Reverse primer（3'-5'）
GbGAPDH	ATCCACGGGAGTCTTCAC	GACCTTCAACAATGCCAAAC
GbCuAO3	AGCATCCTTTGGACCCTCTTAC	TTGGGAGACTACTTCCTGCTTTC
GbPAO13	TGTCTCGTGTCACATTGGGG	GGAAAAAGCCCCATGCACTG
GbPAO6	GTTGCAGGCCTGTGGAAAAG	TCAATGCCTTCCAAGCTCGT
GbPAO8	CTCCCACTGTCCAGAGGAGA	GACATGCCTGCACCTACGAT
GbADC2	GGTGGAGTTTGGGAAGCAGTAT	ACGCCTTGACATAAGAGCTAAGG
GbCuAO1	GAGGTTCCCATGTCGACAGAAT	CAACATCTGCACTCCTGTACCT
GbSPMS2	GGTTCGTCCAGGGATGTTTGTA	TCTCAGACCTCCTCCTGCATAA
